# Multi‐Omics Signatures of Organ Clocks in Biological Aging and Disease: A Conceptual Framework for Organ‐Specific Aging Clocks

**DOI:** 10.1111/acel.70518

**Published:** 2026-04-22

**Authors:** Maria Vasileiou, Gabor Liposits, Bara Barakat, Nam P. Nguyen

**Affiliations:** ^1^ Department of Pharmacy, School of Health Sciences National and Kapodistrian University of Athens Athens Greece; ^2^ Department of Medical Oncology and Haematology Cantonal Hospital St. Gallen, HOCH Health Ostschweiz St. Gallen Switzerland; ^3^ Department of Urology and Robotic Assisted Urology, and Urooncology Hospital Kassel Kassel Germany; ^4^ Department of Radiation Oncology Howard University Washington DC USA

**Keywords:** age‐related diseases, biological aging, chronological age, multi‐omics, organ clocks

## Abstract

Biological aging reflects the progressive decline in cellular and tissue function. Unlike chronological age, biological age is a more accurate indicator of physiological state. Multi‐omics organ clocks have been emerging as promising tools to assess biological aging by integrating genomic, epigenomic, transcriptomic, proteomic, and metabolomic data. These conceptual frameworks suggest that individual organs may age at different rates, explaining variability in the onset and progression of age‐related diseases. However, separate interpretation may overlook the correlation between different omics analyses. A comprehensive, multidimensional analysis is therefore preferred over individual omics for accurate assessment of biological aging. While a comprehensive, multidimensional analysis may provide more holistic insights than single‐omics approaches, the practical implementation of multi‐omics clocks remains limited in clinical settings due to technical differences across omics platforms and dataset availability. This review evaluates current biological clock approaches and explores strategies for multi‐omics integration. By addressing conceptual and methodological gaps, we propose a framework for the development of robust multi‐omics aging clocks.

## Graphical abstract

Organ‐specific aging clocks integrating genomics, epigenomics, transcriptomics, proteomics, and metabolomics offer a more accurate assessment of biological aging than single‐omics approaches. This review proposes a multi‐omics framework emphasizing tissue data, longitudinal study designs, and clinically meaningful training outcomes to advance the development of organ aging clocks.

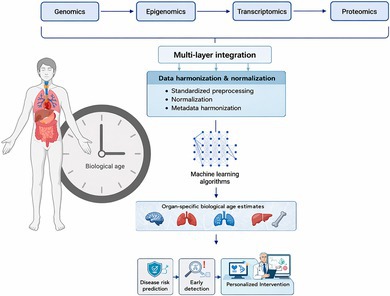

## 1 Introduction

Aging is an inevitable biological process, characterized by a gradual decline in physiological functions, molecular integrity, and an increased susceptibility to chronic diseases. Importantly, biological age does not necessarily correspond to chronological age but instead reflects the physiological state. At a molecular level, aging is a consequence of cellular damage accumulation and the deterioration of protective mechanisms, which together undermine the stability of cells and tissues. Key hallmarks of biological aging include genomic instability, telomere attrition, epigenetic shifts, loss of protein homeostasis, and mitochondrial disruption (López‐Otín et al. 2013). These molecular changes trigger a cascade of reactions, including cellular aging, depletion of stem cells, and changes in intercellular signaling, ultimately compromising tissue function and integrity (Kennedy et al. 2014). Additionally, dysregulated pathways responsible for detecting and responding to nutrients, such as insulin/IGF‐1 signaling and mTOR, play a pivotal role in modulating life duration and aging rates (López‐Otín et al. 2013). With the accumulation of damage in cells, they adopt modified signaling and metabolic states, which contribute to systemic inflammation, commonly referred to as “inflammaging,” thus accelerating age‐related degeneration (Franceschi et al. 2017). While these processes can be captured through individual biomarkers (IL‐6, CRP, and TNF‐alfa), these typically reflect isolated changes on a molecular level rather than on a tissue level. Organ clocks have therefore emerged as integrative models that aggregate molecular patterns to estimate the biological age of individual tissues (Jylhävä et al. 2017).

Biological clocks can be broadly categorized by the outcome used to train them. Early clocks, including Horvath's clock and Hannum's clock, were trained to predict chronological age from DNA methylation patterns and demonstrated the ability to track biological aging across tissues and identify accelerated aging in conditions such as Down syndrome and cancer (Horvath 2013; Hannum et al. 2012). Other clocks have since been developed, providing enhanced predictive accuracy for age‐associated diseases, life expectancy, and personal aging patterns (Fahy et al. 2019). Complementary tools based on transcriptomics, proteomics, and metabolomics are also emerging, providing hence multidimensional insights into aging mechanisms and resilience (Jiang et al. 2021). However, clocks trained on chronological age have generally shown limited ability to predict age‐related disease incidence or mortality in the general population (Rutledge et al. 2022). To address this limitation, a newer generation of clocks has been trained directly on hard outcomes such as all‐cause mortality and disease incidence, DNAm‐based approaches, metabolomics, and routine clinical biomarkers, consistently demonstrating superior performance in predicting health trajectories (Prattichizzo et al. 2024; Rutledge et al. 2022).

Organ‐specific aging clocks differ conceptually from traditional biomarker‐based approaches. While individual biomarkers reflect isolated molecular changes associated with aging, organ clocks integrate coordinated molecular patterns within a specific tissue to estimate its biological age relative to chronological age. This distinction is critical as organs do not age uniformly and may exhibit asynchronous aging trajectories that are not captured by systemic or single‐biomarker models. By quantifying tissue‐level functional decline rather than individual molecular alterations, organ clocks provide a framework for linking molecular aging to organ‐specific disease risk and clinical outcomes.

However, single‐omic approaches provide partial insights due to the multidimensional nature of biological aging which encompasses genetic, epigenetic, transcriptional, proteomic, and metabolic dimensions that interact across tissues and change dynamically over time. Therefore, multi‐omics approaches have gained attention as integrative methods that capture interactions to provide a more holistic assessment of biological aging. Despite these advances, only a few studies have integrated multi‐omics clocks. Even in the case of multi‐omics, technical variability across different omics platforms and limited availability of longitudinal datasets limit the ability to track changes in biological aging. This review aims to evaluate current biological clock approaches, explore strategies for multi‐omics integration, and suggest a respective framework.

## 2 Aging Across Different Omics Modalities

### 2.1 Genomics

Aging is closely linked to a phenomenon known as “genomic convergence”, whereby gene expression profiles become increasingly similar after sexual maturation (Izgi et al. 2022). This convergence suggests that genomic signatures may gradually lose tissue specificity with advancing age, complicating estimates from genomic data alone. As a result, these estimates may reflect systemic aging trends rather than organ‐specific aging.

Genome‐wide association studies (GWAS) have identified multiple genetic variants associated with aging‐related functional decline across different systems. Notably, these studies suggest that younger individuals with impaired organ function may share molecular characteristics with older individuals who exhibit preserved physiological function, underscoring interindividual heterogeneity in aging trajectories (Wheeler and Kim 2011). While GWAS provide valuable insight into genetic contributions to organ dysfunction, they do not constitute genomic organ clocks due to lacking tissue specificity and age‐predictive capacity. Instead, GWAS should be considered as foundational inputs informing risk stratification and potential calibration of downstream organ aging clocks. Key genomic biomarkers implicated in organ‐specific aging are summarized in Table 1.

**TABLE 1:** Key genomic biomarkers for organ‐specific aging.

System	Genomic biomarkers	Aging relevance	Strengths	Limitations
Renal/metabolic system	MMP20, TMPRSS6, ALPL, FADS1	ECM remodeling, fibrosis, and dysregulated iron and lipid metabolism contribute to organ aging (Izgi et al. 2022; Wheeler and Kim 2011).	Tissue vulnerability patterns	Associations may reflect disease rather than intrinsic aging; limited longitudinal data
Nervous system	APOE, TOMM40, CLU, TREM2	Amyloid deposition, cortical atrophy, microglial dysfunction in brain aging and Alzheimer's disease (Yassine and Finch 2020; Yeh et al. 2016)	Strong organ relevance; mechanistic links	Mostly in late‐life disease–driven; limited prediction of biological age earlier in life
Musculoskeletal system	ACTG1, TGFA, SYT1, SLC8A1	Predictors of muscle strength decline, sarcopenia, and age‐related functional decline (Willems et al. 2017).	Functionally relevant to frailty	Sparse longitudinal validation; disease‐specific confounders
Multi‐organ systems	CDKN2B, LPA, HLA‐DQA1	Variants linked to early‐life disease and health span rather than lifespan (Kudryashova et al. 2020).	Health span–lifespan divergence	Limited overlap with established aging markers; underexplored in multi‐omics context
Multi‐organ systems	TP53, RAS, PTEN, TET2	Clonal expansions drive COPD, CVDs, hematologic malignancies; cumulative damage (Vijg and Dong 2020).	Age‐related mutation burden across systems	Highly stochastic; exposure‐dependent; limited predictive accuracy
Multi‐organ systems	Telomere length (TERC, TERT, NAF1, OBFC1, RTEL1)	Shortening limits replicative capacity; genomic stability declines; associated with age‐related disease risk (Shay and Wright 2019; Melzer et al. 2019; Vaiserman and Krasnienkov 2021).	Broad aging relevance; measurable across tissues	Tissue variability; cancer risk trade‐off; requires standardization

Abbreviations: ACTG1, actin gamma 1; ALPL, alkaline phosphatase; APOE, apolipoprotein E; CDKN2B, cyclin‐dependent kinase inhibitor 2B; CLU, clusterin; COPD, chronic obstructive pulmonary disease; CVDs, cardiovascular diseases; ECM, extracellular matrix; FADS1, fatty acid desaturase 1; HLA‐DQA1, human leukocyte antigen DQ alpha 1; LPA, lipoprotein(a); MMP20, matrix metallopeptidase 20; NAF1, nuclear assembly factor 1 ribosome biogenesis homolog; OBFC1, oligonucleotide/oligosaccharide‐binding fold containing 1; PTEN, phosphatase and tensin homolog; RAS, rat sarcoma viral oncogene family; RTEL1, regulator of telomere elongation helicase 1; SLC8A1, solute carrier family 8 member A1; SYT1, synaptotagmin 1; TERC, telomerase RNA component; TERT, telomerase reverse transcriptase; TET2, tet methylcytosine dioxygenase 2; TGFA, transforming growth factor alpha; TMPRSS6, transmembrane serine protease 6; TOMM40, translocase of outer mitochondrial membrane 40; TP53, tumor protein p53; TREM2, triggering receptor expressed on myeloid cells 2.

A recurring observation across tissues is the progressive loss of tissue‐specific gene expression with advancing age, in organs such as the brain, liver, lung, and muscle (Wheeler and Kim 2011). In the nervous system, genes linked to brain aging overlap with neurodegenerative disease pathways suggesting that genomic biomarkers reflect both aging and disease susceptibility (Yassine and Finch 2020; Yeh et al. 2016). Similarly, genes linked to musculoskeletal aging are associated with poor muscle strength (Willems et al. 2017). These genomic signatures highlight organ vulnerability but fall short of capturing aging rates. Although age‐related genes overlap with disease pathways, lifespan and disease incidence are not causally equivalent, as most age‐related diseases manifest later in life (Kudryashova et al. 2020). This distinction highlights the need for genomic frameworks differentiating organ aging driven by intrinsic aging processes from those changes driven by disease.

Additional GWAS have linked somatic and germline mutations to biological aging. Somatic and germline mutations occur due to errors during DNA replication in somatic and germ cells; the former plays a crucial role in the aging process, while the latter leads to genetic diversity and evolution. Somatic mutations are accountable for age‐related diseases such as chronic obstructive pulmonary disease, cardiovascular disease, and hematological malignancies, which are strongly associated with aging. It is important to note that lifestyle (diet, low level of physical activity, and smoking) and environmental factors exacerbate the effects of aging on health with approximately a 25% increase in somatic mutations. The accumulation of de novo somatic mutations inevitably leads to aging and carcinogenic phenotypes (Vijg and Dong 2020). Although somatic mutation burden exhibits organ relevance, it presents major challenges for incorporation into genomic organ clocks, as sampling depth, tissue accessibility, and spatial heterogeneity might affect the accuracy of age estimation. Consequently, somatic mutation profiles currently function as contextual indicators of stress, rather than scalable clock mechanisms for organ aging.

Altogether, these findings indicate that no single genomic biomarker is sufficient to construct a robust organ aging clock. It is important to note that the term “biomarker” is used broadly to encompass several distinct molecular features, including gene variants, somatic mutations, and telomere length. Individual biomarkers provide information on tissue aging risk, but their specificity is limited, as many are also implicated in cancer‐related processes; for instance, telomere shortening and specific somatic mutations (e.g., TP53 and RAS) are prevalent across a wide range of tumors, reducing their utility as aging‐specific markers (Shay and Wright 2019; Melzer et al. 2019; Vaiserman and Krasnienkov 2021). Moreover, most established aging clocks rely on multi‐biomarker models, and genomic features can still contribute as components within multi‐omics frameworks. Rather, a composite genomic panel combined with molecular pathways offers greater predictive value to determine aging rates. From an organ clock perspective, genomics provides static and susceptibility‐based information, whereas temporal and functional resolutions are more effectively captured by transcriptomic and proteomic clocks. Additional genomics limitations should be considered such as the translational relevance of in vitro models, and species constraints of in vivo studies, which may not directly apply to humans due to differences in lifespan and physiology. Thus, genomics is optimally regarded a foundational layer within multi‐omics organ clocks rather than as a standalone tool.

### 2.2 Epigenomics

DNA methylation is a key mechanism that involves the attachment of a methyl group to the fifth carbon atom of a residual cytosine in CpG sites (Chen et al. 2023). Multiple epigenomic clocks have been proposed; while first‐generation models predicting chronological age, second‐generation models predicting biological age. Even though epigenomic clocks vary in the number of CpG sites, they are highly accurate in predicting biological age. Such an example is DunedinPACE, an epigenomic clock using 20 years of longitudinal methylation data to create a novel biomarker (Li et al. 2022). Other examples are PhenoAge and GrimAge which may predict age‐related diseases through methylation patterns alongside biomarkers. These predictions are based on age‐related changes in blood cell composition and leukocyte telomere length. Whereas PhenoAge relies on clinical biomarkers like plasma proteins, GrimAge incorporates surrogate biomarkers (e.g., lifestyle factors) and is considered more effective since surrogate biomarkers outperform clinical ones at mortality prediction. Similarly, AgeAccelGrim utilizes surrogate biomarkers to predict biological instead of chronological age (Levine et al. 2015; Lu et al. 2019; Zhang et al. 2019). Key epigenomic clocks and methylation‐based biomarkers are summarized in Table 2.

**TABLE 2:** Key epigenomic biomarkers for organ‐specific aging.

System	Epigenomic biomarkers	Aging relevance	Strengths	Limitations
Multi‐system	DNA methylation at CpG sites; epigenomic clocks: DunedinPACE, PhenoAge, GrimAge, AgeAccelGrim	Predicts biological age and age‐related disease risk; reflects changes in blood cell composition and leukocyte telomere length (Chen et al. 2023; Li et al. 2022; Levine et al. 2015; Lu et al. 2019; Zhang et al. 2019)	Highly predictive; standardized computational frameworks available	Confounded by tissue specificity, cell composition, and lifestyle factors; limited organ‐level resolution
Nervous system	Histone PTMs (H3.3, H2A.Z), HDACs (Class I–IV), HATs	Changes in chromatin structure, DNA accessibility, and transcriptional control associated with neuronal aging and neurodegeneration (Cao and Dang 2018; Gomez‐Sanchez et al. 2022; Michalak et al. 2019; Maity et al. 2021)	Mechanistic relevance to brain aging; potential therapeutic targets	Complex crosstalk between PTMs; limited longitudinal human data Complex cross‐talk between PTMs; limited longitudinal human data
Multi‐organ systems	RNA modifications: m6A, m5C, A‐to‐I editing; regulators METTL3/14, RNA‐binding proteins	Alters RNA stability, splicing, translation; dysregulation linked to cellular senescence and age‐related disease (Wang et al. 2022)	Captures additional layer of biological aging not reflected in DNA methylation	Emerging field; tissue‐specific patterns not fully mapped; functional consequences incompletely understood

Abbreviations: A‐to‐I, adenosine‐to‐inosine; CpG, cytosine–phosphate–guanine dinucleotide; H2A.Z, histone H2A variant Z; H3.3, histone H3 variant 3; HATs, histone acetyltransferases; HDACs, histone deacetylases; METTL3/14, methyltransferase‐like 3 and 14; m6A, N6‐methyladenosine; m5C, 5‐methylcytosine; PTMs, post‐translational modifications.

In addition to DNA methylation, histone modifications have been implicated in the aging process, yet not incorporated into organ clocks. Post‐translational modifications (PTMs) of histone N‐tails, such as methylation, acetylation, phosphorylation, ubiquitination, and SUMOylation, regulate chromatin structure and gene expression (Cao and Dang 2018). While PTMs are essential for maintaining genomic stability and tissue regeneration, their dysregulation leads to accumulation of DNA damage and neuronal decline (Gomez‐Sanchez et al. 2022; Michalak et al. 2019; Maity et al. 2021). Despite their relevance to aging, histone modifications remain challenging to integrate into epigenomic organ clocks due to tissue accessibility, cell‐type specificity, and technical variability. Similarly, RNA modifications represent an additional layer to epigenomic regulation. Modifications such as N6‐methyladenosine (m6A), N5‐methyladenosine (m5C), and adenosine to inosine (A‐to‐I) editing regulate RNA stability and homeostasis. Dysregulation of these RNA modifications has been associated with cellular senescence and neuronal decline (Wang et al. 2022).

Despite the rapid development of epigenomic clocks, the line between chronological and biological aging remains conceptually blurred, and no universally accepted definition of biological age currently exists. These models estimate biological aging rate in comparison to chronological age, yet several limitations must also be considered, including tissue accessibility, cellular heterogeneity, and the influence of environmental and lifestyle factors on methylation patterns. Critically, the predictive scope of epigenomic clocks lies in the tissue from which they are derived. While Horvath's multi‐tissue clock (2013) was trained across diverse tissue types and captures some degree of tissue specificity, models such as PhenoAge and GrimAge were calibrated on blood‐based biomarkers and therefore exhibit limited resolution for individual organ aging trajectories. Notably, Horvath et al. (2018) demonstrated that the skin and blood clock captures tissue‐specific methylation signatures, suggesting that organ‐specific epigenomic clocks are achievable but require tissue‐appropriate training data. In contrast, most epigenomic clocks are derived from blood samples reflecting systemic rather than organ‐specific aging, which limits their interpretability. Nevertheless, integrating epigenomic clocks with genomic, transcriptomic, and proteomic data can improve organ‐level resolution and enhance predictive capacity within multi‐omics organ clock frameworks.

Overall, epigenomics offers partial organ‐specific information that complements genomics. The tissue of origin is a key determinant of epigenomic clock performance, with blood‐derived clocks capturing systemic aging signals and disease risk, whereas tissue‐derived clocks better reflect organ‐level functional decline. Based on this distinction, future organ‐specific epigenomic clocks should prioritize training on primary tissue samples rather than blood surrogates and should be critically compared against transcriptomic and proteomic approaches to determine which omics layer best captures organ‐aging trajectories. Within multi‐omics organ clocks, epigenomics serves as an intermediate layer linking genetic predisposition to downstream transcriptomic and proteomic changes, thereby enhancing the organ‐specific prediction of biological aging.

### 2.3 Transcriptomics

Transcriptomic analyses provide insight into gene expression changes related to inflammation, cellular senescence, mitochondrial dysfunction, and stress responses, preceding functional decline. Since transcriptomes reflect active gene expression patterns, they capture early changes in biological aging. Representative transcriptomic biomarkers serve as early indicators of biological aging (summarized in Table 3).

**TABLE 3:** Key transcriptomic biomarkers for organ‐specific aging.

System	Transcriptomic biomarkers	Aging relevance	Strengths	Limitations
Nervous system	REST, miRNAs: miR‐21, miR‐34a, miR‐146a; lncRNAs: Airn, pRNA, PINT, HOTAIR	Regulate neuronal gene expression, stress response, chromatin remodeling; contribute to cognitive decline and neurodegeneration^a^	Captures early transcriptional dysregulation and organ‐specific aging; non‐coding RNAs provide mechanistic insight	Tissue‐specific expression; complex regulation; functional impact still emerging; limited longitudinal human data
Musculoskeletal system	ACTG1, TGFA, SYT1, SLC8A1	Predictors of muscle strength decline and sarcopenia (Willems et al. 2017)	Functionally relevant; direct link to frailty	Mostly disease‐associated; limited longitudinal validation
Multi‐organ systems	Pro‐inflammatory and senescence‐associated secretory phenotype (SASP) genes (TNF‐α, IL‐1, IL‐6, CDKN1A, CDKN2A)	Chronic low‐grade inflammation (inflammaging) and senescence‐associated secretory phenotype (SASP) accelerate systemic aging (Franceschi et al. 2018; Li et al. 2023; Songkiatisak et al. 2022; Emelyanova et al. 2018; Kumari and Jat 2021; Saul et al. 2022; Hellmich et al. 2023; Zhang et al. 2022)	Provides early‐warning signals of functional decline across multiple systems	Highly context‐dependent; inflammatory status influenced by environment, infection, and lifestyle
Multi‐organ system	Mitochondrial and stress‐response genes	Reflects mitochondrial dysfunction, redox imbalance, and cellular senescence (Emelyanova et al. 2018; Kumari and Jat 2021)	Integrates metabolic and stress‐response changes; applicable across tissues	Expression variability across cell types; correlation with functional decline may be direct

Abbreviations: ACTG1, actin gamma 1; Airn, antisense imprinted RNA in the Igf2r locus; CDKN1A, cyclin‐dependent kinase inhibitor 1A (p21); CDKN2A, cyclin‐dependent kinase inhibitor 2A (p16); HOTAIR, HOX transcript antisense RNA; IL‐1, interleukin 1; IL‐6, interleukin 6; lncRNAs, long non‐coding RNAs; miR‐21/34a/146a, microRNA 21/34a/146a; miRNAs, microRNAs; PINT, p53‐induced noncoding transcript; pRNA, promoter‐associated RNA; REST, RE1‐silencing transcription factor; SASP, senescence‐associated secretory phenotype; SLC8A1, solute carrier family 8 member A1; SYT1, synaptotagmin 1; TGFA, transforming growth factor alpha; TNF‐α, tumor necrosis factor alpha.

^a^ Bashir et al. (2023); Mampay and Sheridan (2019); Varesi et al. (2023); Tarkhov et al. (2019); Kogan et al. (2015); Franceschi et al. (2018); Li et al. (2023); Songkiatisak et al. (2022); Emelyanova et al. (2018); Kumari and Jat 2021; Saul et al. (2022); Hellmich et al. (2023); Zhang et al. (2022); Ugalde et al. (2013); Kim et al. (2018); Boon et al. (2013); Gong et al. (2022); Picerno et al. (2022).

In contrast to isolated transcriptomic biomarkers, transcriptomic clocks integrate coordinated gene expression patterns to estimate the biological age of a tissue (Tarkhov et al. 2019; Kogan et al. 2015). Conceptually, they may offer advantages for organ aging assessments in certain contexts, as they are responsive to local microenvironmental cues and, when trained on tissue‐specific data, have the potential to reflect organ‐specific functional states more directly, including immune activation, metabolic adaptation, and stress resilience (Franceschi et al. 2018; Li et al. 2023; Songkiatisak et al. 2022; Emelyanova et al. 2018). However, potential advantages must be weighed against key limitations: transcriptomic data are inherently more variable than DNA methylation data, with higher noise levels driven by environmental factors, post‐transcriptional regulation, and cell‐type compositional shifts. Indeed, tissue‐specific epigenomic clocks such as the skin and blood clock (Horvath et al. 2018) demonstrate that DNA methylation can also capture tissue‐specific aging signatures with high reproducibility, even though many established epigenomic clocks are derived from blood and thus reflect systemic processes. Overall, neither omics layer is universally superior; rather, their complementary strengths support integrated multi‐omics approaches, especially given challenges such as tissue accessibility, cellular heterogeneity, and transcriptome variability across individuals and time points (Kumari and Jat 2021; Saul et al. 2022; Hellmich et al. 2023).

Most transcriptomic aging models have primarily been derived from bulk tissue or blood, which measure gene expression as an aggregate signal from multiple cell types, limiting their applicability to organ‐specific assessments. Nevertheless, transcriptomics provides insights into gene networks that can be combined with epigenomic and proteomic data. As transcriptomic technologies advance, transcriptomic organ clocks may emerge as powerful tools for linking molecular trajectories to tissue‐specific functional decline.

### 2.4 Proteomics

Considering that proteins represent the functional output of gene expression, proteomic clocks are particularly well suited for organ‐specific aging assessments. By capturing tissue‐level physiological changes, proteomic clocks provide a closer link between molecular aging processes and organ function than upstream omics layers. While some studies have argued that proteomic approaches more directly reflect the phenotype compared to genomic, transcriptomic, and epigenomic biomarkers since they directly reflect on the phenotype (Kliuchnikova et al. 2024), it is important to note that several validated aging clocks, including the Horvath clock, GrimAge, and PhenoAge, are based on DNA methylation. Being closer to phenotype does not inherently confer predictive superiority, as stability, reproducibility, and longtidual validation are equally critical parameters. The strength of proteomics may therefore depend on the specific organ system, outcome of interest, and the availability of tissue data. It is evident that integrating signals across multiple omic layers can provide a robust framework for organ‐specific aging clocks. Early proteomic clocks based on plasma or cerebrospinal fluid leveraged aptamer‐based platforms to quantify target proteins and predict biological age (Menni et al. 2014; Baird et al. 2012). Johnson et al. (2020) proposed a proteomic clock comprised of 23 proteins, which may be extended to 85 proteins for more robust results. Large cohort studies confirmed that both the shortened and extended versions of this proteomic aging clock could predict human age. However, this proteomic clock relied on several different techniques, which limits detection and might omit several proteins. Nevertheless, they managed to identify a large number of proteins that are linked to aging (Johnson et al. 2020). Recently, Kuo et al. (2024) developed an advanced proteomic clock, the proteomic aging clock (PAC), which is able to predict all‐cause mortality. Unlike previous models, PAC has high predictive power as it leverages the largest dataset of proteins and individuals in the world (Kuo et al. 2024). Proteomic clocks find application in cardiovascular, respiratory, and neurodegenerative diseases, furthermore in psychological disorders, as analyzed below (Baird et al. 2012; Johnson et al. 2020; Kuo et al. 2024; Diniz et al. 2016).

Proteomic clocks leverage proteomic biomarkers to capture key aging processes including inflammation, atherosclerosis, and metabolic dysfunction, as described in Table 4 (Perry et al. 2023; Cotter et al. 2015; Brundage et al. 2024; Wischhusen et al. 2020; Herder et al. 2017; Yang et al. 2023; Egbuche et al. 2020). These proteomic biomarkers manifest in organ‐specific changes, such as extracellular matrix (ECM) remodeling in the heart, linked to cardiac dysfunction and fibrosis through mTOR signaling and SASP pathways (Silva et al. 2021; Santinha et al. 2024; Tanaka et al. 2020; Levi et al. 2020). In the central nervous system, proteomic biomarkers have been used to estimate brain age, capturing neuroinflammation, myelination, synaptic function, mitochondrial activity, and amyloid processing that underline neurodegenerative diseases (Casanova et al. 2024; Rivera et al. 2022; Gu et al. 2025; Sasaki et al. 2021; Mohamedi et al. 2020; Wingo et al. 2019; Quinn et al. 2021). Brain aging is closely connected to age‐related ocular disorders such as cataract, glaucoma, and age‐related macular degeneration (AMD), which share overlapping proteomic patterns with neurodegenerative disorders, such as ECM remodeling, amyloid deposition, and metabolic reprogramming, highlighting common aging pathways (Pathai et al. 2013; García‐Quintanilla et al. 2022; Senabouth et al. 2022; Guo et al. 2023; Korb et al. 2024; Nordestgaard et al. 2021). At the systemic level, proteomic biomarkers capture inflammaging and immunosenescence; the former is characterized by low‐grade chronic inflammation, ECM degradation, fibrosis, and oxidative stress, which are not fully captured by DNA methylation clocks (Franceschi et al. 2018; Argentieri et al. 2024; Teissier et al. 2022; Vatic et al. 2020; Martens et al. 2021; Bakun et al. 2013; Nkuipou‐Kenfack et al. 2015). A comprehensive multidimensional analysis integrating proteomics along with genomic, epigenomic, and transcriptomic datasets is therefore essential for accurate assessment of biological aging (Wu et al. 2022). A comparative summary of the strengths, limitations, and roles of each omics layer is presented in Table 5.

**TABLE 4:** Key proteomic biomarkers for organ‐specific aging.

System	Proteomic biomarkers	Aging relevance	Strengths	Limitations
Cardiovascular system	GDF‐15, NT‐proBNP, IL‐1RA, FABP‐4, ECM proteins (COL6A6, VTN, MFGE8), STC‐1, IGFBPs, TIMPs	Involved in inflammation, fibrosis, ECM remodeling, and SASP; predict cardiovascular aging and frailty (Perry et al. 2023; Cotter et al. 2015; Brundage et al. 2024; Wischhusen et al. 2020; Herder et al. 2017; Yang et al. 2023; Egbuche et al. 2020; Silva et al. 2021; Santinha et al. 2024; Tanaka et al. 2020; Levi et al. 2020)	Directly reflects tissue‐level functional decline; clinically measurable	Proteomic assays vary in sensitivity; longitudinal validation limited
Nervous system	EGF, HSPA1B, MMPs, ADAMTS, VGF, SEPT5, DBI, MAPT, PHF24	Influences neurodegeneration, amyloid processing, synaptic function; predicts brain aging (Casanova et al. 2024; Rivera et al. 2022; Gu et al. 2025; Sasaki et al. 2021; Mohamedi et al. 2020; Wingo et al. 2019; Quinn et al. 2021)	High organ‐specific relevance; captures early cognitive decline	Cohort variability; protein abundance influenced by systemic factors; limited organ accessibility
Ocular system	TF, APOA1, C3, LCN1	Linked to age‐related macular degeneration (AMD) and overlaps with neurodegeneration (Pathai et al. 2013; García‐Quintanilla et al. 2022; Senabouth et al. 2022; Guo et al. 2023; Korb et al. 2024; Nordestgaard et al. 2021)	Reflects common pathways between retinal and brain aging	Limited to specialized tissues; accessibility for repeated measures is difficult
Multi‐organ systems	TNF‐α, IL‐1, IL‐6, CRP, UPP signature, IgA‐1, IgG‐3, LAIR1, collagen fragments	Captures inflammaging, immunosenescence, and ECM turnover (Argentieri et al. 2024; Teissier et al. 2022; Vatic et al. 2020; Martens et al. 2021; Bakun et al. 2013; Nkuipou‐Kenfack et al. 2015)	Provides systemic aging signal; measurable in blood/urine	Highly variable; influenced by lifestyle, infection, and disease; temporal dynamics complex
Multi‐organ systems		Combines multiple tissue‐level changes to reflect biological age; correlates with genomics, epigenomics, transcriptomics (Baird et al. 2012; Johnson et al. 2020; Kuo et al. 2024; Diniz et al. 2016; Wu et al. 2022)	Captures holistic biological aging; supports multi‐omics integration	Integration complex; data normalization and platform variability pose challenges

Abbreviations: ADAMTS, a disintegrin and metalloproteinase with thrombospondin motifs; APOA1, apolipoprotein A1; COL6A6, collagen type VI alpha 6 chain; CRP, C‐reactive protein; DBI, diazepam binding inhibitor; ECM, extracellular matrix; EGF, epidermal growth factor; FABP‐4, fatty acid binding protein 4; GDF‐15, growth differentiation factor 15; HSPA1B, heat shock protein family A (Hsp70) member 1B; IGFBPs, insulin‐like growth factor binding proteins; IL‐1, interleukin 1; IL‐1RA, interleukin 1 receptor antagonist; IL‐6, interleukin 6; LAIR1, leukocyte associated immunoglobulin like receptor 1; LCN1, lipocalin 1; MAPT, microtubule‐associated protein tau; MFGE8, milk fat globule‐EGF factor 8; NT‐proBNP, *N*‐terminal pro–B‐type natriuretic peptide; PHF24, PHD finger protein 24; SEPT5, septin 5; STC‐1, stanniocalcin 1; TF, transferrin; TNF‐α, tumor necrosis factor alpha; UPP, urinary proteomic profile; VGF, VGF nerve growth factor inducible; VTN, vitronectin.

**TABLE 5:** Comparative overview of omics approaches for organ‐specific aging clocks.

Parameter	Genomics	Epigenomics	Transcriptomics	Proteomics
Tissue specificity	Low: genomic signatures lose tissue specificity with age	Moderate: blood‐derived clocks reflect systemic rather than organ‐level aging; tissue‐derived clocks offer greater specificity	High: responsive to microenvironmental cues when trained on primary tissue	High: captures tissue‐level functional changes
Temporal resolution	Static: reflects fixed genetic susceptibility	Moderate: methylation accumulates over time	Dynamic: reflects real‐time gene expression	Dynamic: reflects real‐time functional output of gene expression
Signal stability	High: genetic variants are stable through time and tissues	High: methylation patterns are reproducible and robust	Low: interindividual variability across time points and environmental conditions	Moderate: influenced by assay sensitivity and sample
Limitations	Longitudinal resolution; tissue accessibility	Organ‐level resolution; lifestyle and environmental factors	High noise levels; cell heterogeneity	Assay sensitivity; limited longitudinal validation
Role	Foundational: provides genetic predisposition context	Bridging: links genetic predisposition to expression	Readout: captures active states of organ‐specific decline	Functional: directly captures tissue function and phenotype

## 3 Discussion and Future Perspectives

This review highlights the emerging potential of multi‐omics organ clocks in characterizing biological aging. Genomic biomarkers indicate genomic instability, while epigenetic modification, such as DNA methylation and histone changes, translate these genetic predispositions into tissue‐level changes. Transcriptomics captures actively expressed genes, whereas proteomics reflects resulting biological processes such as inflammation, mitochondrial dysfunction, and metabolic reprogramming that characterize tissue‐specific aging. In this context, emerging tools provide complementary perspectives, enabling a holistic view of aging processes. A direct cross‐modal comparison reveals important differences in tissue specificity, noise, and predictive scope across omics layers. Genomics provides static susceptibility information with low signal‐to‐noise but poor temporal resolution. Epigenomics, particularly DNA methylation, offers high reproducibility and moderate tissue specificity when tissue‐appropriate training data are available, but most current clocks are derived from blood and capture systemic rather than organ‐level signals. Transcriptomics has higher tissue specificity potential but lower signal stability due to transcriptome variability across individuals and time points. Proteomics provides the closest approximation to tissue phenotype but is limited by assay sensitivity, cohort variability, and sample accessibility. A summary of these omics categories and their integration within the proposed framework is presented in Table 6. While individual omics approaches provide valuable insights, their integration is essential for a comprehensive and accurate assessment of biological aging and its link to diseases. Multi‐organ proteomic clocks illustrate this potential, with Wen (2025) showing that integrating proteomic data and phenotypic data improves biological age estimation and uncovers genetic overlaps with disease endpoints, highlighting the translational power of multi‐omics frameworks for predicting organ‐specific health trajectories (Wen 2025). Incorporating advanced computational methods, such as machine learning algorithms and network‐based modeling, can improve predictive accuracy and reveal complex interactions underlying organ aging.

**TABLE 6:** Summary of omics categories with regards to organ clocks and their integration within the proposed framework.

Omics category	Biomarkers	Proposed integration approach
Genomics	Single nucleotide polymorphisms (SNPs)	Foundation for multi‐omics models to understand how changes in one omics layer affect others.
	Telomere length	
	Somatic mutations	
Epigenomics	DNA methylation sites	Combine with transcriptomic and proteomic data to identify networks that regulate age‐related gene expression in specific organs.
	Histone modifications	
	Chromatin structure	
Transcriptomics	mRNA, lncRNA, and miRNA expression profiles	Combine with epigenomic and proteomic data to identify pathways behind organ aging.
Proteomics	Post‐translational modifications	Align with transcriptomic data to link gene expression patterns with molecular signatures and aging phenotypes.

Organ clocks reveal the biological age of individual organs that may differ significantly from their chronological age, potentially explaining the heterogeneity observed in age‐related diseases. For instance, epigenomic aging in the cardiovascular system has been associated with early signs of atherosclerosis, while proteomic aging in the brain may correlate with early onset of neurodegenerative diseases. Multi‐omics organ clocks could serve as early warning systems, supporting prevention strategies, monitoring, and personalized interventions for age‐related diseases. In the long‐term, organ clock assessments may be included in clinical evaluations, along with personalized lifestyle changes, monitoring, and therapeutic interventions. However, their integration and application in routine clinical practice are currently constrained by several practical challenges.

The integration of multi‐omics faces several important obstacles. These include technical differences across omics platforms, limited longitudinal datasets, and population‐specific biases. Omics platforms may vary in technical aspects, such as sample processing and data normalization. These variations can affect the accuracy of aging estimates in the general population. Furthermore, the absence of longitudinal datasets hinders the ability to track changes over time. It should be noted that biological aging is an inherently longitudinal process, yet most organ clock studies rely on cross‐sectional biomarker measurements. Recent evidence demonstrates that molecular changes do not necessarily increase monotonically with chronologically age, highlighting the importance of repeated measures to capture non‐linear trajectories (Pusparum et al. 2025). Additionally, population characteristics, such as age, ethnicity, lifestyle, and environmental factors, make it difficult to generalize these estimates to different populations. Although predictive models can identify correlations between genomic or molecular changes and biological age, establishing a causal link remains a challenge (Teschendorff and Horvath 2025). Addressing these issues requires the development of standardized protocols for sample handling, data normalization, and quality control. These efforts should be supported by longitudinal and multi‐ethnic cohort studies integrating computational tools to establish causality of predictive models.

Beyond these issues, the practical implementation of multi‐omics integration introduces complexity, as each omics layer operates at vastly different dimensional scales. Genomics may involve millions of SNPs, epigenomics hundreds of thousands of CpG sites, transcriptomics thousands of genes, and proteomics hundreds to thousands of proteins, introducing substantial dimensionality mismatch. This dimensional mismatch requires careful feature selection and integration strategies to avoid overfitting and ensure interpretability. In practice, several computational approaches have been proposed to address these issues. Multi‐omics factor analysis (MOFA) identifies common patterns across omics layers, helping to separate aging‐related signals from signal noise. Sparse canonical correlation analysis (sCCA) links features across modalities, such as methylation changes and protein levels. Network‐based methods model interactions across omics layers, and ensemble machine learning approaches combine omics layer predictions into a unified aging score, providing predictive value.

Tissue specificity and dataset heterogeneity further exacerbate integration challenges, as signals from different tissues may not be directly comparable. An overlooked issue is the methodological heterogeneity in how individual features are assigned to specific organs across different studies. Approaches vary widely, from general biological knowledge to expression thresholds derived from the Human Protein Atlas or GTEx, whereby a feature is considered organ‐enriched only if it meets a minimum expression threshold in the target tissue relative to others. For instance, Oh et al. (2023) required proteins to be expressed at least four times higher in one organ than the other, using GTEx data. Other studies use different thresholds or rely on tissue specificity score, meaning that the same protein may be assigned to different organs depending on the approach. This lack of harmonization inevitably affects which features are included in each clock and limits comparability across studies. Future frameworks should therefore standardize organ assignment criteria to ensure reproducibility and cross‐study comparability.

**FIGURE 1:**
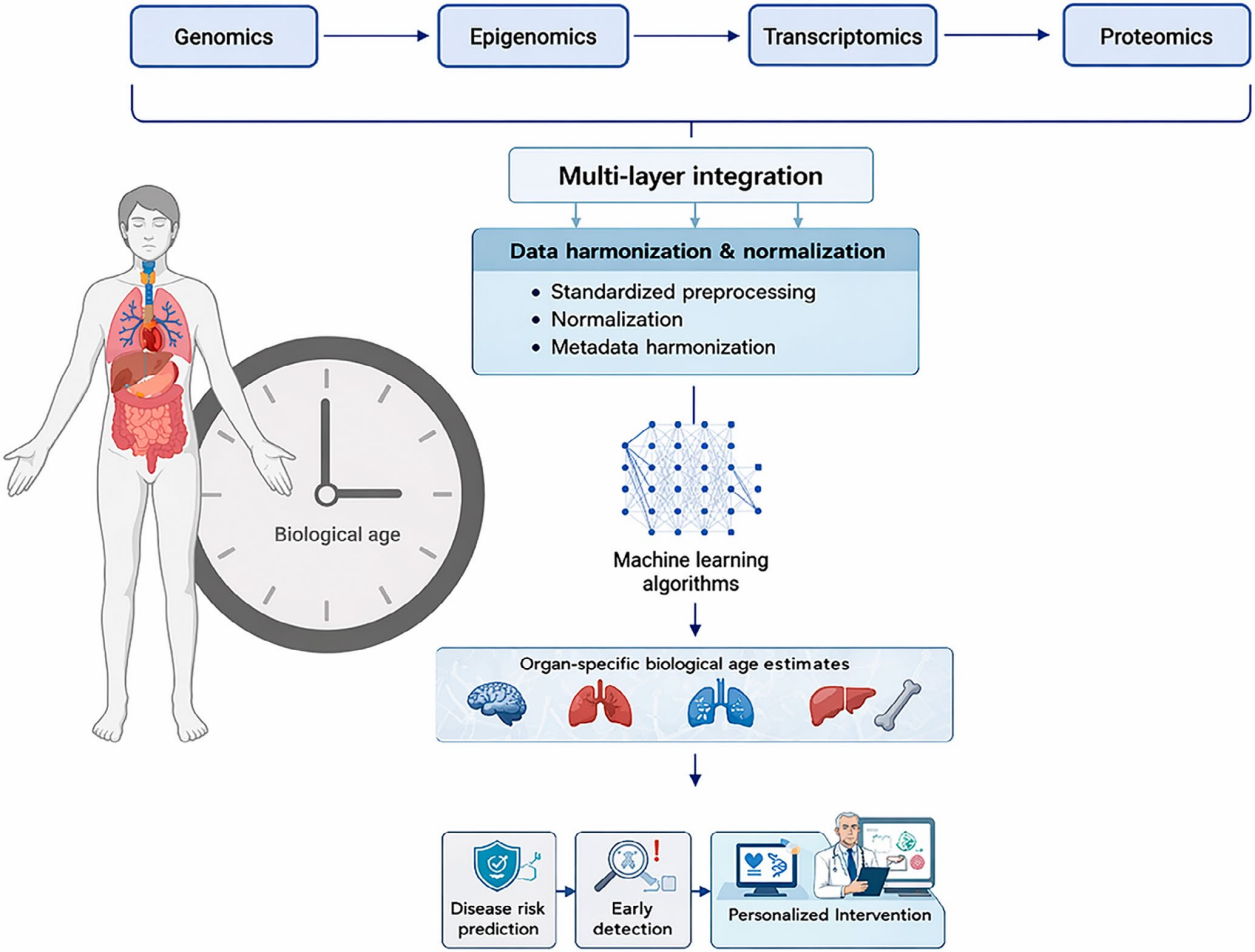
Multi‐omics framework for organ‐specific aging clocks. The framework illustrates the integration of multiple omics layers—genomics, epigenomics, transcriptomics, proteomics, and metabolomics—into organ‐specific biological age estimates. Each omics layer data is harmonized and integrated using machine learning algorithms to generate organ‐specific biological age estimates. Machine learning algorithms are then applied to the harmonized data to generate organ‐specific biological age estimates for the brain, heart, lung, liver, and musculoskeletal system. The clock icon represents biological age acceleration, reflecting the deviation of an organ's biological age from its chronological age. These organ‐specific estimates are translated into three clinical applications: disease risk prediction, early detection, and personalized intervention.

To address these limitations, future research should focus on integrating multi‐omics—combining genomics, epigenomics, transcriptomics, and proteomics—along with standardized methodologies and larger, more diverse datasets, as illustrated in Figure 1. Beyond these established omics layers, clocks derived from clinical biomarkers and metabolomic signatures represent modalities that are currently underrepresented in the literature on organ‐specific aging. In this regard, Pusparum et al. (2025) compared multiple biological clock data from the same individuals measured over time, demonstrating that different clock types capture distinct and complementary aspects of aging, and that biological age does not increase monotonically with chronological age. These findings underscore the value of longitudinal, multi‐modal designs and the need for integrative frameworks. Expansion to multi‐population cohorts will make aging models more generalizable and less subject to demographic or lifestyle factors. These efforts should be supported by multi‐institutional collaborations that share high‐quality real‐world data, establish analytical frameworks and ensure protocol harmonization. Their integration with imaging‐based omics or digital pathology can potentially uncover organ aging differences and fully interpret age‐related diseases.

### 3.1 Multi‐Omics Framework for Organ‐Specific Aging Clocks

The proposed framework addresses the current conceptual and methodological gaps by emphasizing three aspects; data harmonization, multi‐layer integration, and translational validation. By applying this framework, researchers can move beyond catalogs of biomarkers toward predictive models of organ aging.

### 3.2 Data Harmonization

Normalization and cross‐platform calibration need to be standardized to ensure comparability among omics datasets. This includes harmonizing preprocessing pipelines, batch effect corrections, and scaling procedures thus measurements from different platforms or laboratories can be meaningfully compared. Harmonized metadata, including demographics, tissue origin, and disease status, will improve reproducibility and allow estimates to the general population. Additionally, consistent annotation of technical variables, such as sequencing depth or assay type, and systematic recording of quality control metrics are essential. By implementing these measures, datasets can be reliably integrated, enabling cross‐study analyses and enhancing the generalizability of these findings.

### 3.3 Multi‐Layer Integration

Integration of multiple omics layers requires advanced computational approaches capable of handling high‐dimensional, heterogeneous data. Machine learning approaches can be utilized to generate genomic, epigenomic, transcriptomic, and proteomic models. Specifically, techniques such as multi‐omics factor analysis (MOFA), Bayesian inference, and deep learning can be applied to identify signatures reflecting biological age.

A critical component of multi‐layer integration is feature selection and dimensionality reduction; these isolate the most informative molecular signatures while avoiding overfitting. Incorporating network‐based analyses can reveal interactions between different omics features, highlighting pathways behind organ‐specific aging. Cross‐validation on independent cohorts and integration with public multi‐omics repositories are essential to enhance model generalizability. Most importantly, sensitivity analyses should be conducted to assess the robustness of organ clock predictions, different integration strategies, and omics combinations.

### 3.4 Translational Validation

Achieving clinical utility will require longitudinal validation in diverse populations. These datasets should include a wide range of ages, ethnicities, lifestyle, environmental factors, and underrepresented populations to minimize bias and ensure broad applicability. Validation should include correlations with established clinical outcomes, imaging markers, and measures of organ health. By integrating multi‐omics signatures with clinical phenotypes, researchers can identify biomarkers predictive of disease onset, progression, and response to intervention. Prospective interventional studies where modifiable risk factors are monitored alongside organ clocks provide opportunities for early detection and personalized preventive strategies. Establishing metrics will facilitate the acceptance and adoption of organ clocks in clinical practice. It is evident that integrating multi‐omics datasets with imaging and clinical biomarkers will enhance translational significance. This approach will enable the development of personalized diagnostics and targeted interventions for age‐related diseases.

## 4 Conclusion

Organ clocks have transformed our understanding of age‐related diseases by providing valuable insights into tissue‐specific aging and therapeutic approaches. This review highlights that no single omics layer is sufficient to capture the full complexity of organ‐specific biological aging, and that integrating genomics, epigenomics, transcriptomics, proteomics, and metabolomic is essential for accurate and generalizable clock development. Overall, the predictive value of organ clocks depends on three interconnected factors: the source of training data, the choice of clinical outcome, and the study design. Key priorities for future research include clocks trained on primary tissue data rather than blood surrogates, validated against clinically meaningful outcomes such as mortality or disease incidence, and built on longitudinal rather than cross‐sectional designs are most likely to capture true organ‐level aging trajectories and translate into clinical practice. Moving forward, standardizing organ criteria across the same individuals over time will allow study reproducibility and comparability. Achieving this requires not only methodological advances but also collaborative efforts through data sharing, expansion of longitudinal cohorts, and clinical validation across diverse populations.

## Author Contributions

Maria Vasileiou: conceptualization, writing – original draft, writing – review and editing; Gabor Liposits: writing – review and editing; Bara Barakat: writing – review and editing; Nam P. Nguyen: writing – review and editing, supervision. All authors reviewed and approved the final version of the manuscript.

## Funding

The authors have nothing to report.

## Ethics Statement

The authors have nothing to report.

## Conflicts of Interest

The authors declare no conflicts of interest.

## Data Availability

Data sharing not applicable to this article as no datasets were generated or analysed during the current study.
